# A novel approach for rapid screening of mitochondrial D310 polymorphism

**DOI:** 10.1186/1471-2407-6-21

**Published:** 2006-01-24

**Authors:** Cenk Aral, Handan Kaya, Çiğdem Ataizi-Çelikel, Mustafa Akkiprik, Özgür Sönmez, Bahadır M Güllüoğlu, Ayşe Özer

**Affiliations:** 1 Marmara University, School of Medicine, Department of Medical Biology, Tıbbiye Cad. No.49, 81326, Haydarpasa, Istanbul, Turkey; 2 Marmara University Hospital, Department of Pathology, Tophanelioglu C. Kosuyolu, Uskudar, Istanbul, Turkey; 3 Marmara University Hospital, Department of Surgery, Tophanelioglu C. Kosuyolu, Uskudar, Istanbul, Turkey

## Abstract

**Background:**

Mutations in the mitochondrial DNA (mtDNA) have been reported in a wide variety of human neoplasms. A polynucleotide tract extending from 303 to 315 nucleotide positions (D310) within the non-coding region of mtDNA has been identified as a mutational hotspot of primary tumors. This region consists of two polycytosine stretches interrupted by a thymidine nucleotide. The number of cytosines at the first and second stretches are 7 and 5 respectively, according to the GeneBank sequence. The first stretch exhibits a polymorphic length variation (6-C to 9-C) among individuals and has been investigated in many cancer types. Large-scale studies are needed to clarify the relationship between cytosine number and cancer development/progression. However, time and money consuming methods such as radioactivity-based gel electrophoresis and sequencing, are not appropriate for the determination of this polymorphism for large case-control studies. In this study, we conducted a rapid RFLP analysis using a restriction enzyme, BsaXI, for the single step simple determination of 7-C carriers at the first stretch in D310 region.

**Methods:**

25 colorectal cancer patients, 25 breast cancer patients and 41 healthy individuals were enrolled into the study. PCR amplification followed by restriction enzyme digestion of D310 region was performed for RFLP analysis. Digestion products were analysed by agarose gel electrophoresis. Sequencing was also applied to samples in order to confirm the RFLP data.

**Results:**

Samples containing 7-C at first stretch of D310 region were successfully determined by the BsaXI RFLP method. Heteroplasmy and homoplasmy for 7-C content was also determined as evidenced by direct sequencing. Forty-one percent of the studied samples were found to be BsaXI positive. Furthermore, BsaXI status of colorectal cancer samples were significantly different from that of healthy individuals.

**Conclusion:**

In conclusion, BsaXI RFLP analysis is a simple and rapid approach for the single step determination of D310 polymorphism of mitochondrial DNA. This method allows the evaluation of a significant proportion of samples without the need for sequencing- and/or radioactivity-based techniques.

## Background

Human mitochondrial DNA (mtDNA) is composed of a 16.6 kb, double stranded, circular DNA molecule that encodes 13 polypeptides of the respiratory chain, 22 transfer RNAs and 2 ribosomal RNAs [1]. Mitochondrial DNA alterations have been suspected to play an important role in the development and progression of cancer. Several mutations have been identified in a wide variety of human tumors, including breast, colorectal, ovarian, gastric, hepatic and esophageal cancers, as well as hematological malignancies [2-6]. D-loop region of the mtDNA is the most potent accumulation site for many of these mutations and numerous polymorphisms have also been reported in this region. This is explained by the lack of protective histones, high oxidative stress and deficient DNA repair mechanisms [5]. D-loop is the only non-coding mtDNA region which contains crucial elements for replication and transcription. Thus, the sequence alterations of this region may contribute to altered replication or transcription properties [4,5].

Recently, Sanches-Cespedes and colleagues have identified a polyC mononucleotide repeat located between 303 and 315 nucleotides within the D-loop region as a mitochondrial hot spot of deletion or insertion mutations [7]. This region is a part of the conserved sequence block (CSB) II and consists of a stretch of cytosines interrupted by a thymine nucleotide (CCCCCCCTCCCCC). Although the number of cytosine residues at the first stretch of polyC is accepted as 7-C (GeneBank NC_001807), it is highly polymorphic ranging between 6-C to 9-C [1,8-10]. It is still questionable whether there is any correlation between the number of cytosine residues and the development and/or progression of cancer. Typically, time and money consuming methods such as sequencing and radioactivity-based gel electrophoresis are required in order to evaluate this polymorphism among individuals. Moreover, gel electrophoresis remains ineffective unless confirmed with sequencing. Especially in large population studies, these limidations become obviously important.

The aims of this study were to develop a restriction fragment length polymorphism (RFLP) assay for the single step rapid screening of individuals that carry first stretch 7-C at mitochondrial D310 region and to evaluate if any difference exists among healthy individuals and cancer patients in the Turkish population. We tested a total of 141 tissue samples including normal and cancerous tissues of 25 breast and 25 colorectal cancer patients and 41 blood samples of healthy individuals. By applying this simple approach without a need for sequencing and/or radioactive labelling, 41% of the studied samples were found to have 7-C in D310 region. Furthermore, we compared the cases and normal samples for their RFLP status and found a statistically significant difference between colorectal cancer samples and healthy individuals.

## Methods

### Tissue specimens and DNA extraction

Paraffin embedded tissue specimens from primary tumors and matched normal (adjacent non-neoplastic) tissues were selected from 25 breast cancer and 25 colorectal cancer patients treated at Marmara University Hospital between 1990–2003. Briefly, 6 μm thick sections were cut from blocks that had been selected for maximal tumor content. These sections from formalin-fixed paraffin-embedded tissue blocks were deparaffinized first by washing with xylene, then with absolute ethanol. The deparaffinized tissues were incubated at 55°C in 150 μL of digestion buffer (0.5 mg/ml proteinase K; 0.05 M Tris-HCl, pH 8.5; 1 mM EDTA; 0.5% Tween 20) for three hours. Then the enzyme was inactivated by heating for 10 minutes at 94°C, and the samples were centrifuged at 12,000 *g *for 10 minutes. The supernatant containing total genomic DNA was aliquoted and stored at -20°C [11]. Control DNA from blood samples of healthy volunteers was also extracted by proteinase K digestion followed by phenol/chloroform extraction according to John et al [12]. The study protocol was approved by the Marmara University Faculty of Medicine Research Ethics Committee.

### Genotyping assays of D310 repeat

D310 region of mtDNA was amplified by polymerase chain reaction (PCR) as described by Sanches-Cespedes et al [7]. The primer sequences were as follows: forward 5-ACAATTGAATGTCTTGCACAGCCACTT-3 and reverse 5-GGCAGAGATGTGTTTAAGTGCTG-3. PCR amplications were performed in a 50 μl volume containing 200 μM of each dNTP, 12.5 pmol of each of the forward and reverse primers, 50 mM KCl, 10 mM Tris-HCl (pH 9.0), 1.5 mM MgCl_2 _and 1 U of Taq DNA polymerase (Fermentas, Lithuania). PCR cycling conditions were as follows: 96°C for 90 s, followed by 40 cycles of 96°C for 30 s, 60°C for 30 s and 72°C for 30 s, then extension at 72°C for 5 min (Biometra, UNO-Thermoblock model of thermocycler, Germany). 109 bp PCR products were checked by 1.8% agarose gel electrophoresis.

PCR products were subjected to restriction enzyme digestion. 5 μl of PCR product was mixed with 3 U of BsaXI (New England Biolabs, USA) in 1× buffer containing 50 mM potassium acetate, 20 mM tris-acetate, 10 mM magnesium acetate, 1 mM DTT, pH 7.9. The samples were incubated at 37°C for 19 hours to ensure complete digestion. Restriction products were separated on 4% agarose gel. Samples were also processed for DNA sequencing using forward primer by fluorescence-based automatic sequence analyzer (ABI PRISM 310, USA).

Data comparison was made by chi-square test. *p *< 0.05 was considered as statistically significant.

## Results

**Figure 1:**
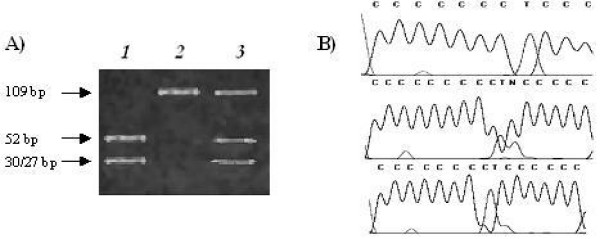
A) Agarose gel electrophoresis of BsaXI digestion of 109 bp D310 region of 3 colorectal cancer patients. Lane 1: BsaXI (+) Lane 2: BsaXI (-) Lane 3: Heteroplasmic B) Respective sequence analysis profiles: *The upper*: BsaXI (+) homoplasmy with 7-C at the first stretch; *the middle*: BsaXI (-) with 8 and 9-C heteroplasmy; *the lower*: Heteroplasmic for BsaXI digestion with 7 and 8-C.

We conducted an RFLP assay in order to determine 7-C carrying individuals in mtDNA D310 region by eliminating the time-consuming sequencing method. We amplified D310 region and then exposed it to digestion with BsaXI (Fig 1). The recognition sequence of the restriction enzyme is ACNNNNNCTCC. If the sequence of the first stretch of D310 region consists of 7-C, digestion occurs and two bands appear on the agarose gel corresponding to 52 bp and 30/27 bp, referred as BsaXI positive (Fig 1A, lane 1). A single band corresponding to 109 bp PCR product is seen at the gel for BsaXI (-) cases (Fig 1A, lane 2). In this state, number of cytosine residues is either more or less than 7-C. At the heteroplasmic state, all of those bands (109, 52, 30/27 bp) appear (Fig 1A, Lane 3). In order to confirm this analysis, automated fluorescence based sequencing was also applied and results of 3 cases are given in Fig 1B. As seen in this figure, BsaXI digestion was consistent with sequence analysis. It must be noted that this approach allows only the determination of first stretch of polyC with 7-C. Homoplasmic 7-C sequence of BsaXI positive samples was confirmed by automated sequence analysis (Fig 1B, upper panel). On the other hand, the BsaXI negative sample (Fig 1A, lane 2) has polymorphic variants containing 8-C and 9-C in its mtDNA pool (Fig 1B, middle panel). Finally, heteroplasmy for BsaXI (Fig 1A, lane 3) showed that the patients have both 7-C and 8-C sequences (Fig 1B, lower panel).

Table 1 shows BsaXI RFLP status of 25 colorectal and 25 breast carcinoma samples with their surrounding normal tissues and of blood samples from 41 healthy individuals tested. In case of the breast cancer samples, 12 of 25 (48%) cases were BsaXI positive, whereas 11 patients were BsaXI negative (44%) and 2 (8%) were heteroplasmic (Table 1). On the other hand, heteroplasmy was not encountered in corresponding healthy tissue samples. Except for the two heteroplasmic cases, all of the breast cancer samples showed the same digestion profile as their corresponding healthy tissues. In these heteroplasmic cases, one of the normal tissues surrounding the tumor tissues was BsaXI positive and the other was negative. Cases with different BsaXI status in tumor and its corresponding normal tissues are shown in Table 2.

Regarding colorectal tumor samples, 9 out of 25 cases (36%) were BsaXI positive and 10 cases (40%) were BsaXI negative. Twenty-four percent (6/25) of the cases were heteroplasmic for BsaXI digestion (Table 1). As seen in the breast cancer samples, colorectal tumor samples had almost the same digestion profile with that of the matched normal tissues. On the other hand, three heteroplasmic and one homoplasmic BsaXI positive tumor samples showed different patterns than corresponding normal tissue (Table 2). The tumor counterparts of two BsaXI positive and one BsaXI negative normal tissues are heteroplasmic. In the BsaXI positive tumor sample, the corresponding normal tissue showed BsaXI negative genotype (Table 2). Differences between tumor samples and corresponding normal tissues were not statistically significant in either colorectal or breast cancer tissues (*p *> 0.05). Forty-one healthy individuals (10 men and 31 women) were also tested for D310 BsaXI RFLP analysis. No heteroplasmy was shown in these individuals. BsaXI positive and negative cases were 34.1% (14/41) and 65.9% (27/41), respectively (Table 1). We also compared BsaXI status of the samples of patients with control samples from healthy patients. Interestingly, when control group was compared with the colorectal tumor group, there was a statistically significant difference for BsaXI status (p = 0.003). However, BsaXI negative cases had lower frequency in the breast cancer samples (11/25, 44%) when compared to the control group (27/41, 65.9%), but this difference was not statistically significant (p= 0.063). It should be noted that males were excluded when comparing the breast cancer cases with the healthy individuals to make a gender match between the cases and controls.

## Discussion

Recently, researchers have focused on mtDNA D-loop alterations in a variety of human tumors. In the first description by Sanches-Cespedes et al., the D310 region of the D-loop was studied by a simple PCR based approach following a radioactivity-based denaturating polyacrylamide gel electrophoresis or direct sequencing [7]. The authors reported that 22% of primary tumor samples showed D310 first polyC stretch alterations. Parrella et al. reported sequence alterations in 19% of the breast cancer samples and showed also the same alterations in matched lymph node or fine needle aspirated samples [9]. Various percentages of D310 alterations were also reported by several researchers in studies on thyroid, breast, cervical and gastric cancers [10,13-15].

Radioactivity-based gel electrophoresis is widely used for the determination of D310 first stretch polymorphism, but this method has two important limitations. First of all, the method can only detect the difference between patients and control individuals, but the exact cytosine number at the first stretch cannot be clarified unless confirmed by sequencing. Secondly, the use of radioactive compounds makes this method inappropriate for large population studies. Although sequencing is a gold standard for mutation research, it is a laborious and time-consuming method. Here, we tested a novel rapid pre-screening RFLP method that may be useful for studies with relatively large number of cases. As expected, a significant number of cases could be determined for the D310 first stretch status (41%) without need for sequencing approach. Thus, by using this approach, a significant percentage of cases can be determined for their cytosine number at the first stretch of D310 region and the remaining cases may be further analysed by sequencing.

Although the relationship between BsaXI RFLP status and tumor development is not clear, in this study a statistically significant difference was found between colorectal cancer patients and healthy individuals. Moreover, there was no significant difference between tumor samples and matched normal tissues for BsaXI RFLP status. Cytosine numbers may be different for BsaXI negative cases. Since the digestion profile of a 6-C, an 8-C or a 9-C stretch are the same, the method applied in this study allows to determine the exact cytosine number only when it is 7.

In conclusion, determination of the BsaXI RFLP status of D310 region of D-loop of mtDNA is a new, simple, cost-effective and promising approach and can be applied for the screening of large populations.

## Competing interests

The author(s) declare that they have no competing interests.

## Authors' contributions

AÖ and CA have equally contributed to this work. MA and ÖS have carried out DNA isolation and quantitation and were involved in the collection of samples. HK, ÇAÇ and BMG were involved in the collection and clinical characterization of tumor samples.

## Pre-publication history

The pre-publication history for this paper can be accessed here:



## Figures and Tables

**Table 1:** Summary of BsaXI RFLP data.

	**Breast cancer**		**Colorectal cancer**		**Healthy individuals**
	*n: 25*		*n: 25*		*n: 41*
	*Mean age: 59.5 ± 13.8*		*Mean age: 62.4 ± 10.6*		*Mean age: 59.2 ± 11.9*
	
	Tumor (n)	Control (n)	Tumor (n)	Control (n)	(n)
**Bsa XI (+)**	12	13	9	10	14
**BsaXI (-)**	11	12	10	12	27
**Heteroplasmy**	2	0	6	3	0

**Table 2:** Cases with different BsaXI status in tumor and corresponding normal tissues.

**Tumor type**	**Patient ID**	**BsaXI Status**	
		
		**Tumor**	**Normal**
**Breast cancer**	1712	Heteroplasmy	BsaXI (-)
	2955	Heteroplasmy	BsaXI (+)
**Colorectal cancer**	116	BsaXI (+)	BsaXI (-)
	779	Heteroplasmy	BsaXI (-)
	1619	Heteroplasmy	BsaXI (+)
	5367	Heteroplasmy	BsaXI (+)
